# Classification of Microglia in Human Alzheimer’s Disease Postmortem Cases

**DOI:** 10.1002/alz.089604

**Published:** 2025-01-03

**Authors:** Terri‐Leigh Stephen, Kenneth Nguyen, Debra Hawes, Bayla M Breningstall, Michael S Bienkowski

**Affiliations:** ^1^ USC, Los Angeles, CA USA; ^2^ University of Southern California, Los Angeles, CA USA; ^3^ Stevens Neuroimaging and Informatics Institute, Keck School of Medicine, University of Southern California, Los Angeles, CA USA

## Abstract

**Background:**

Microglia undergo varying regional dependent functional changes, which can exacerbate cognitive decline in Alzheimer’s disease, but the full clinical relevance remains unclear. Ramified microglia survey the micro‐environment and inert/amoeboid microglia engulf debris. A third morphological type; rod microglia, have been observed in a number of pathological conditions, but are relatively understudied. It has been suggested that they form after acute neurological insult and understanding their anatomical distribution across brains with Alzheimer’s disease may provide useful information regarding disease progression.

**Method:**

University of Southern California Alzheimer’s Disease Research Center (USC ADRC) post‐mortem hippocampal and prefrontal cortical sections were immunostained with 4G8 for amyloid, AT8 for neurofibrillary tau tangles and Iba1 for microglia and imaged using an Olympus VS120 slide scanning microscope. 20 Alzheimer’s disease cases and 10 control cases were selected to best represent population demographics (balanced for sex and ApoE genotype). We utilized Ilastik machine learning toolkit to classify amyloid pathology (dense, diffuse, and amyloid precursor protein), neurofibrillary tangles, neuritic plaques and microglia (rod, inert and ramified) within stained tissue section images. These classifications were then overlaid within manually segmented region maps (aligned with the Allen Human Brain Atlas) using the Quantitative Imaging Toolkit (QIT).

**Result:**

Our findings revealed elevated rod and inert microglial staining in Alzheimer’s disease cases compared to controls alongside an increase in pathology burden across disease stage. Ramified microglia were reduced in Alzheimer’s disease cases compared to controls. Iba1 was correlated with amyloid pathology, specifically in the dentate gyrus (DG) of the hippocampus. Rod microglia were significantly more elongated with more processes in Alzheimer’s disease cases compared to controls. In general ramified had more processes, inert tended to be smaller with rod falling in between.

**Conclusion:**

Regional variations in microglia morphology and function in Alzheimer’s disease could reflect differences in Alzheimer’s disease cell type susceptibility and have implications for understanding the disease progression.

